# Overexpression of steroid sulfotransferase genes is associated with worsened prognosis and with immune exclusion in clear cell-renal cell carcinoma

**DOI:** 10.18632/aging.102392

**Published:** 2019-10-27

**Authors:** Yuqing Li, Qiang Ding, Zuquan Xiong, Hui Wen, Chenchen Feng

**Affiliations:** 1 Department of Urology, Huashan Hospital, Fudan University, Shanghai 200040, PR China

**Keywords:** clear cell-renal cell carcinoma, steroid sulfotransferase, cancer immunity

## Abstract

Aim: Steroid sulfotransferase (SULT) plays physiological roles but its role in clear cell-renal cell carcinoma (ccRCC) remains unclear. We therefore investigated genetic alteration of steroid SULT genes in ccRCC.

Results: Overexpression of any of SULT genes occurred in ~8% of ccRCC patients. Overexpression of steroid SULT genes was associated with worsened prognosis. Steroid SULT gene-upregulated ccRCC cases showed mutual exclusivity with mutations of VHL, SETD2 and PBRM1, and with focal deletions of 3p and 9p, respectively. Expressions of SULT genes were negatively correlated with that of VHL, SETD2 and PBRM1, respectively. While no cancer-intrinsic pathway was enriched, immune signatures were significantly enriched in SULT gene-overexpressed cases, resulting in significantly fewer infiltration of lymphocytes. Targeting SULT1B1 significantly inhibited growth of ccRCC cells.

Conclusion: Steroid SULT genes were associated with worsened prognosis and with immune exclusion in ccRCC.

Methods: In silico reproduction of TGGA and GTEx datasets was performed. Data were processed comprehensively using the platforms of cBioPotal, GEPIA, Human Protein Atlas, TIMER, respectively. Functional annotation was analyzed using platforms of NET-GE and GSEA, respectively. In vitro assays were performed for validation.

## INTRODUCTION

Renal cell carcinoma (RCC), the most common type of kidney cancer, originates from the lining of the proximal convoluted tubule. As the seventh most common malignant cancer in men and ninth in women, RCC is reported to cause 102.000 deaths per year [1]. The clear cell renal cell carcinoma (ccRCC) is the most common type of RCC and accounts for 90% of the entity. Despite satisfactory cure rate by surgical removal, patients with localized ccRCC still face a recurrence rate at 20%-30%. Moreover, 30% of newly diagnosed cases have metastatic lesions [2].

Sulfotransferases (SULT) are enzymes that catalyze the sulphonation. SULT can transfer a sulfo group from a donor molecule to an acceptor alcohol or amine, participate in plenty of important metabolic activities on protein, lipid, carbohydrate or steroid in cytoplasm and membrane [3, 4]. Sulfotransferase family contains 64 members in human and is divided into several types based on the structure and function. Of the 16 cytoplasmic sulfotransferases, SULT1A1, SULT1E1, SULT2A1 and SULT2B1 are clustered and defined as steroid sulfotransferases that catalyze steroid metabolism [5].

Interplay between SULT genes and cancer has drawn much attention recently. A recent study demonstrates that overexpression of HS3ST2, 3B and 4 in breast cancer are associated with c-Src, Akt and NF-κB, showing common tumor-promoting activity [6]. 34324q `123 disease-free and overall survival in patients with pancreatic ductal adenocarcinoma [7]. However, little is known about expression of SULT genes and their prognostic role in ccRCC.

In the current study, we aim to demonstrate expressions and prognostic contribution of SULT genes in ccRCC. With reproduction of multiple genetic and genomic datasets, we strive to provide pilot evidence for understanding biologic activity and therapeutic potential of steroid SULT genes in ccRCC.

## RESULTS

### Overexpression of steroid SULT genes is associated with worsened prognosis in ccRCC

**Figure 1:**
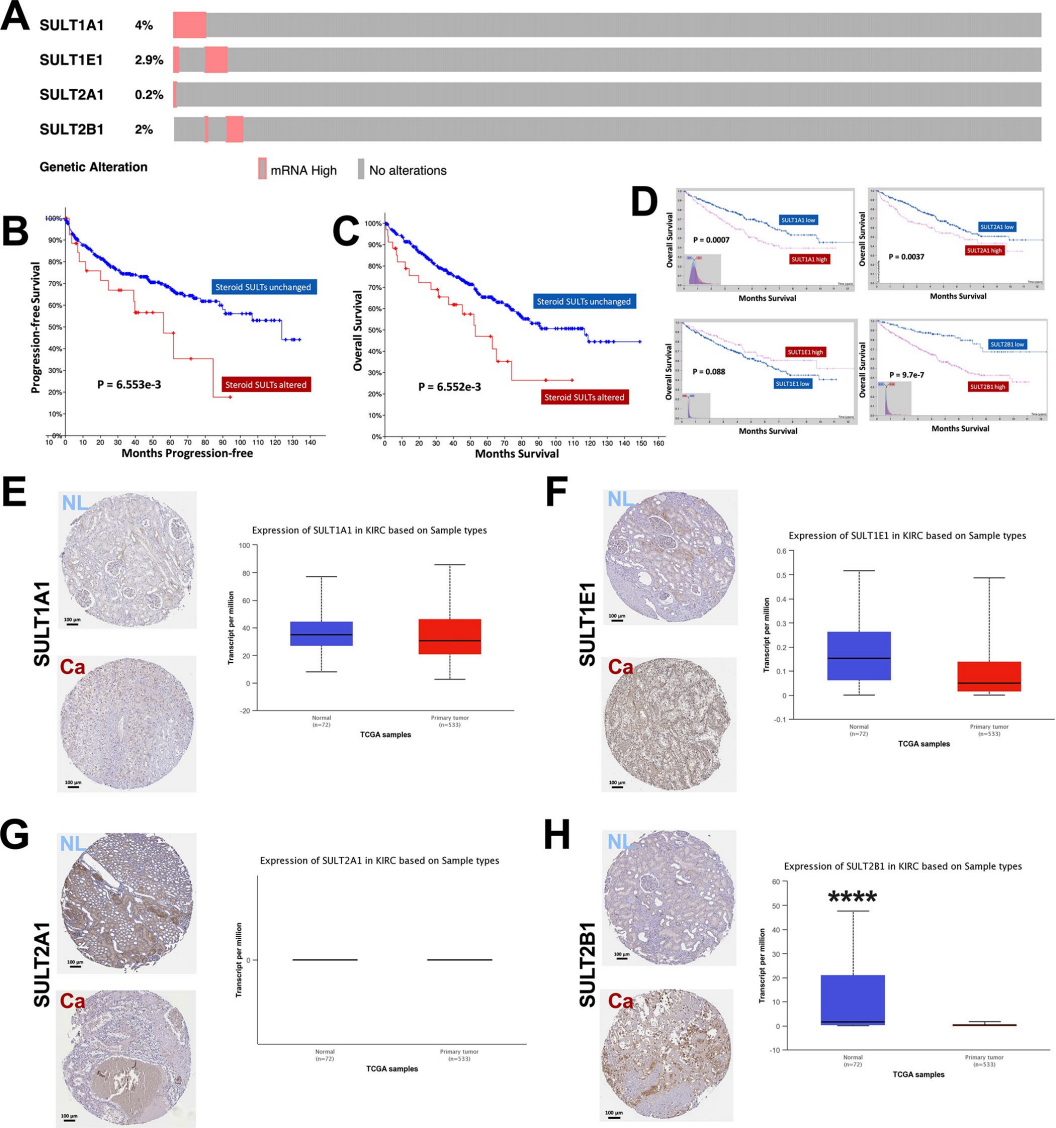
Overexpression of steroid SULT genes is associated with worsened prognosis in ccRCC. Reproduced from the TCGA KIRC dataset with cBioPortal platform, shown are (**A**) Percentage of ccRCC case with overexpression of steroid sulfotransferase (SULT) genes amongst 534 cases; (**B**–**C**) Progression-free and overall survival (OS) between ccRCC patients with steroid SULT genes upregulated and unchanged. Reproduced from TCGA KIRC dataset with Human Protein Atlas platform, shown are (**D**) OS of ccRCC patients with high or low mRNA level of SULT1A1, SULT1E1, SULT2A2 and SULT2B1 at automatically defined cutoffs. Reproduced from TCGA KIRC dataset with GEPIA and UALCAN platform, shown are (**E–H**) Contrast in gene expressions of steroid SULT genes between normal and cancer tissue, respectively (****P < 0.0001).

We first analyzed alteration type of steroid SULT genes in ccRCC and found that overexpression was the sole type of alteration in cases (Figure 1A). Forty-four cases (8%) presented overexpression of any of the steroid SULT genes (Figure 1A). On Oncoprint, overexpression of the genes showed trend for mutual exclusivity indicating functional non-redundancy between the family members. Despite only 8% of cases showed alteration, patients with upregulated steroid SULT genes had significantly worsened progression-free and overall survival compared with those without (Figure 1B–1C). We then investigated prognostic value of each gene. As cases with overexpression was too few to generate statistical significance, we used expressional cutoff to evaluate prognostic role of each gene. We found that higher expressions of SULT1A1, SULT1A2, and SULT2B1 were significantly associated with worsened prognosis whilst higher SULT1E1 expression only showed a trend (Figure 1D). In reminiscence of that SULT1E1 was overexpressed in only 1 case who also harbored overexpressed SULT1A1 and SULT1A2 (Figure 1A), those findings further supported functional non-redundancy of the genes. We then investigated whether steroid SULT genes were differentially expressed between normal and caner tissue of kidney by integrating data from TCGA. We found that SULT1A1 and SULTE1 were expressed at similar levels in cancer as compared to normal kidney tissue (Figure 1E–1F). Interestingly, SULT2A1 mRNA was neither detected in normal nor cancer tissue in TCGA in line with nonspecific IHC staining shown in HPA (Figure 1G). This could be due to limited cases in HPA cohort given that only 1 case showed overexpression of SULT2A1 in TCGA cohort. SULT2B1 showed substantial higher expression in kidney cancer as compared to normal tissue, which was corroborated by IHC staining (Figure 1H). Here we showed that steroid SULT gene-upregulated ccRCC represented a poor phenotype despite alteration rate at ~8%.

### Functional enrichment of steroid SULT gene-altered ccRCC

**Figure 2:**
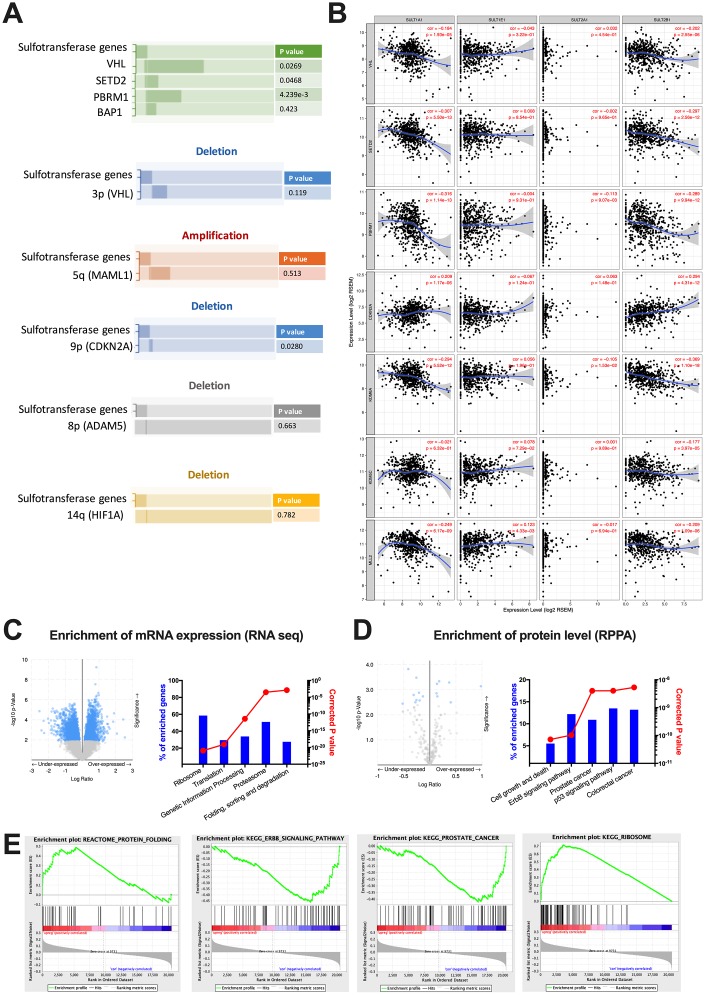
**Functional enrichment of steroid SULT gene-altered ccRCC.** Reproduced from the TCGA KIRC dataset with cBioPortal platform, shown are (**A**) Pattern and significances of enrichment of significantly mutated genes and frequent copy number variances indicated by hallmark genes between cases with upregulated or unchanged steroid SULT gene. (**B**) Correlation of expression level between steroid SULT genes and VHL, SETD2, PBRM1, KDM6A, KDM5C, MLL2 and CDKN2A. (**C**–**D**) functional analysis of enriched genes at mRNA (RNA seq) and protein levels (RPPA) generated at cBioPortal (volcano plots on left) and processed at NET-GE platform (bar plots on right) and (**E**) Select enriched gene sets were validated using the GSEA enrichment analyses.

By far, there is only a dearth of studies unveiling roles of SULTs in cancer, let alone in ccRCC. Previous mechanistic reports on physiological roles of SULTs provided little insights into its oncogenic roles. We therefore performed enrichment analyses at mutation, copy number, mRNA expression and protein levels. We found that alteration of steroid of SULT genes was mutually exclusive with mutations of VHL, SETD2, and PBRM1, respectively, but not with that of BAP1 (Figure 2A). This corresponded to the mutual exclusive mutation pattern between PBRM1 and BAP1 [8]. We then queried hallmark genes on 3p, 5q, 9p, 8p and 14q to capture arm-level alterations [9]. We found that neither 3p loss nor 5q gain showed mutual exclusivity or co-occurrence, whereas loss of 9p showed significant mutual exclusivity with steroid SULT gene alteration (Figure 2A). No correlation was found for 8p and 14q deletion. We then further studied expression correlations of individual SULT gene with that of hallmark genes of ccRCC. We showed significant negative correlations between expressions of SULT1A1 and VHL, SETD2, PBRM1, KDM6A, KDM5C and MLL2 respectively (Figure 2B). Similar pattern was also observed for SULT2B1 (Figure 2B). Those results indicated SULT gene-upregulated cases were characterized with strong 3p suppression despite not via co-existing mutation or focal arm deletion but at expression level. Though partial correlation reveal significant correlations between expressions of SULT1A1, SULT2B1 and CDKN2A, the sigmoid curve indicated lack of biological correlation (Figure 2B). Functional annotation was first done by performing enrichment on cBioPortal and submitting genes passing FDR to net-based enrichment analysis. As shown, top 5 enriched annotations both at mRNA level non-specific (Figure 2C). The top 5 functional annotation at protein level, however, yielded three more specific pathways like cell growth and death, ERBB signaling and p53 signaling (Figure 2D). However, established knowledge of biology indicated the latter 2 signaling relatively inactive in ccRCC. We therefore preformed enrichment validation using an alternative method. Using GSEA, we validated all enriched pathways shown in NET-GE platform and found none of the findings above reached FDR q value of < 0.05 (Figure 2E). So far we have not yet identified cancer intrinsic pathways that provided biological explanation for SULT gene-associated prognostic phenotype and we thus aimed at cancer immunity, which was prominent in ccRCC.

### Steroid SULT genes were associated with immune exclusion in ccRCC

**Figure 3:**
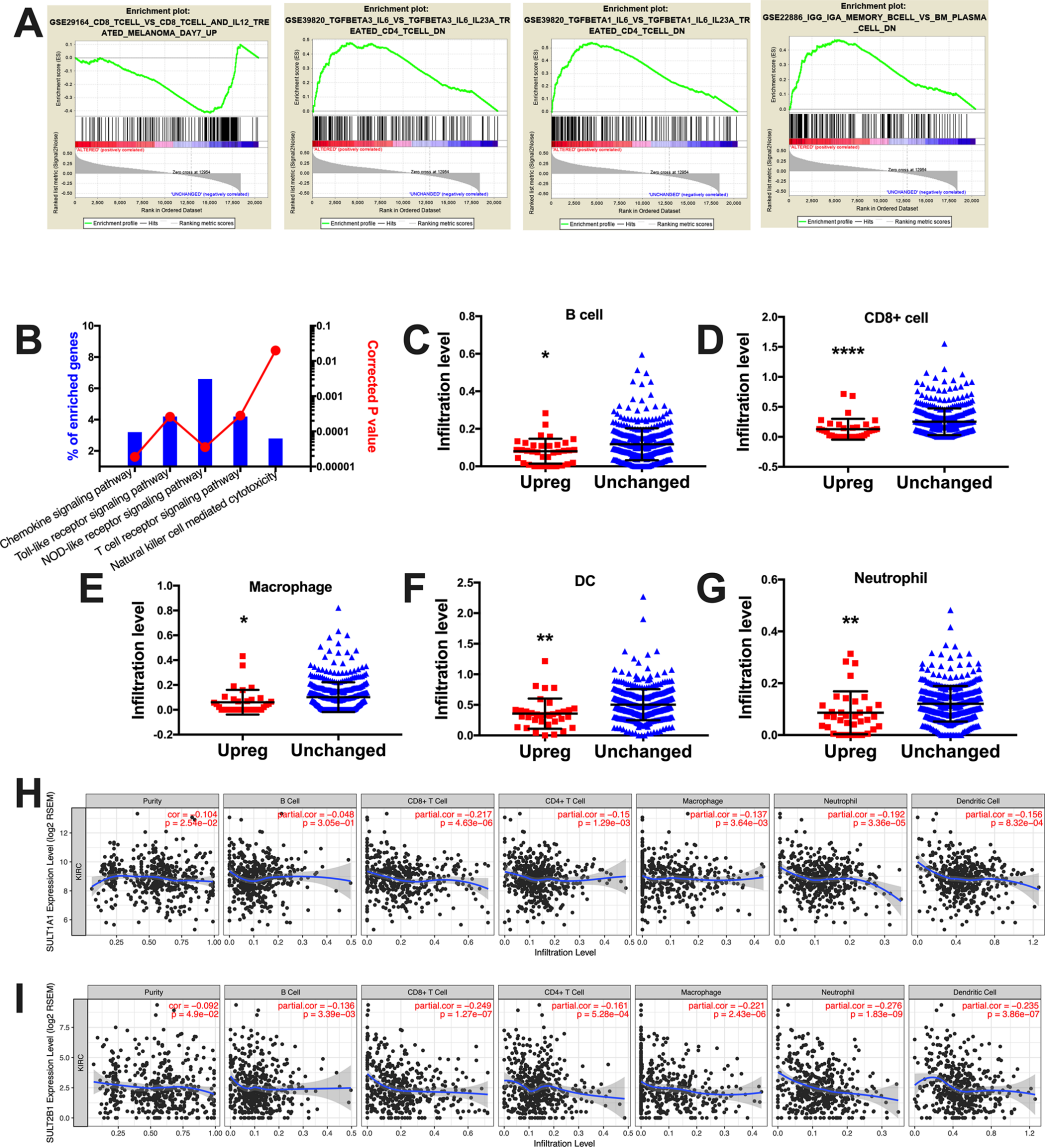
**Steroid SULT genes were associated with immune exclusion in ccRCC.** (**A**) Select enriched genesets validated via the GSEA enrichment analyses. (**B**) Immune analysis of enriched genes processed at NET-GE platform (bar plots on right). (**C–G**) Correlation between immune infiltration and cases with steroid SULT genes upregulated and unchanged. (**H–I**) Correlation between expression of individual steroid SULT gene and immune infiltration.

When GSEA analysis was performed using the immune signatures, we noticed 735 gene sets enriched in SULT gene-upregulated cases (Figure 3A). By further mining the protein enrichment by NET-GE, we noticed significantly enriched pathways within immune response (Figure 3B). Combining both findings, we could pinpoint signatures for increased immune stimulus in SULT gene-unchanged cases and for exclusion of immune response in SULT gene-upregulated cases (Figure 3A). Using the TIMER platform, we studied immune infiltrate levels between cases with upregulated or unchanged SULT gene expressions and found that upregulated cases showed significantly lower levels of B cells, CD8+ cells, macrophages, dendritic cells, and neutrophil cells, where has CD4+ cells remained unchanged (Figure 3C–3G). We then tried to identify contribution of individual SULT gene to immune infiltration. We found correlation could only be established for SULT1A1 and SULT2B1. For both genes, expressions were not negatively correlated with tumor purity indicating genes were expressed predominantly on tumor cells (Figure 3H–3I). For SULT1A1, significant negative correlation was noted for CD8+ cells, CD4+ cells, macrophages, and neutrophils (Figure 3H). For SULT2B1, significant negative correlation was noted for B cells, CD8+ cells, CD4+ cells, macrophages, neutrophils, and dendritic cells (Figure 3I). Here we showed that steroid SULT genes were associated with immune exclusion pathway and correspondingly with decreased tumor infiltrating lymphocytes.

### Targeting SULT2B1 inhibits ccRCC in vitro

**Figure 4:**
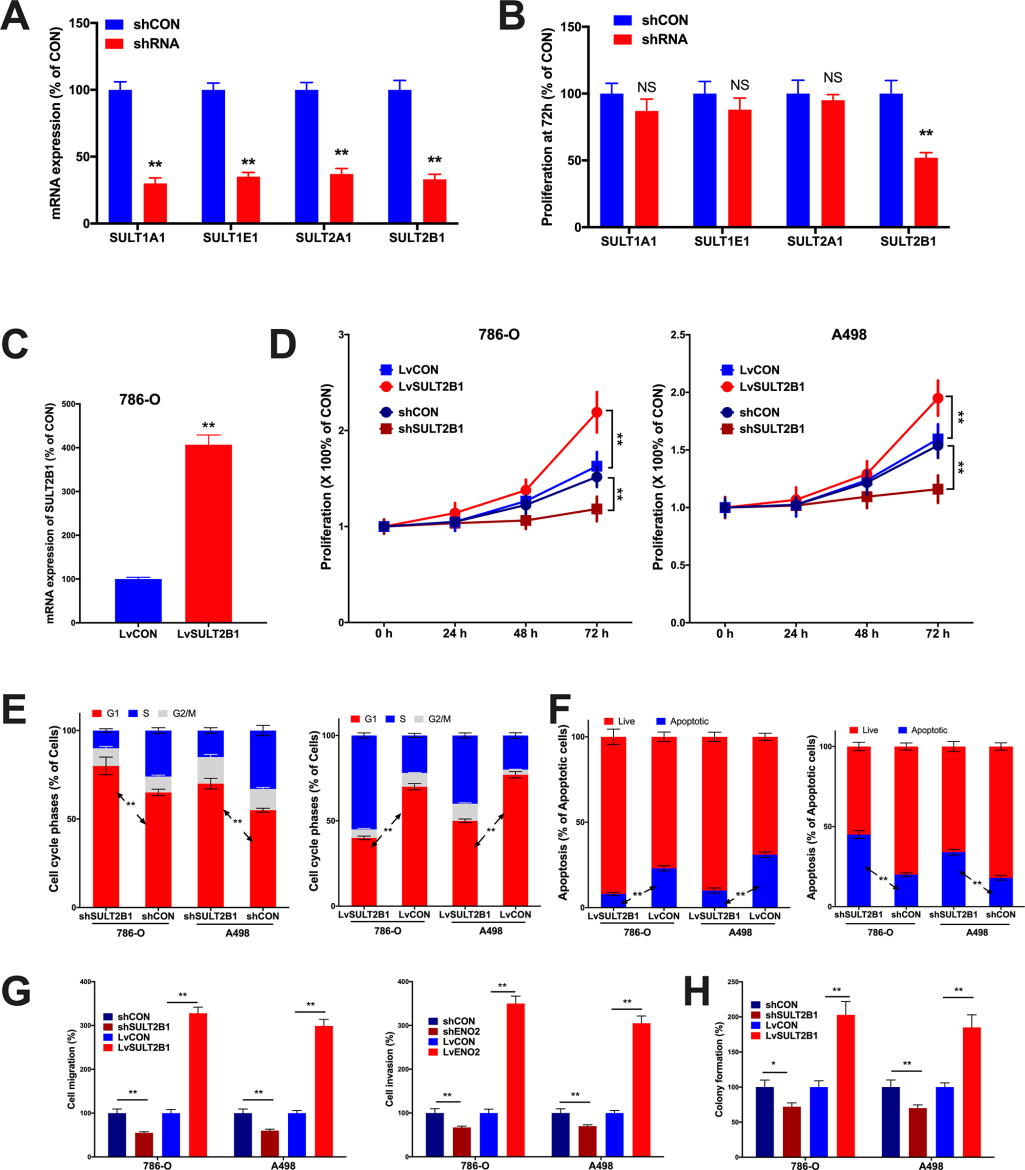
**In vitro assays showing functional analysis of SULT genes in ccRCC.** (**A**) shown are efficacies of shRNAs targeting SULT genes in 786-O cells; (**B**) Proliferation at 72 hours of culture detected by MTT assay in 786-O cells; (**C**) shown was efficacy of lentiviral delivery of SULT1B1 overexpression; Shown were overexpression and silencing of SULT2B1 impacting on (**D**) proliferation by MTT, (**E**) cell cycle population and (**F**) apoptosis by flow cytometry, (**G**) migration and invasion by Transwell assay, and (**H**) colony formation assay (n = 4, *P < 0.05; **P < 0.01).

In vitro assays were performed to validate pro-tumorigenic role of SULTs. By silencing the 4 SULT genes, respectively, we found that SULT2B1 silencing significantly inhibited proliferation of 786-O cells (Figure 4A). We then applied SULT2B1 overexpression (Figure 4C) to both 786-O and A498 ccRCC cell lines and found that SULT2B1 overexpression significantly increased proliferation in both cell lines (Figure 4D). SULT2B1 silencing significantly increased population in G1 phase and SULT2B1 overexpression significantly decreased population in G1 phase in both cell lines (Figure 4E). SULT2B1 silencing significantly decreased apoptosis and SULT2B1 overexpression significantly increased apoptosis (Figure 4F). Transwell assays showed SULT2B1 silencing significantly decreased invasion and migration in both cell lines and SULT2B1 overexpression significantly increased invasion and migration (Figure 4G) as well as anchorage-independent growth profiled by colony formation assay (Figure 4H). Here we validated in part the SULT2B1 exerted pro-tumorigenic role in ccRCC, in line with our findings in silico.

## DISCUSSION

The sulfotransferases contain a variety of enzymes that are cellular-functionally divided into several classes, one of which is soluble sulfotransferase catalyzing sulfation of molecules like phenols and estrogens. Another class, called membrane anchored sulfotransferase, catalyzes the sulfation of carbohydrates and tyrosyl residues of larger proteins or peptides [10]. The sulfotransferases function when transferring a sulfo group from 3′-phosphoadenylyl sulfate to the hydroxyl group of an acceptor. Such progress is related to endogenous compounds (such as steroids and bile acids), dietary constituents (such as flavonoids) and some drugs. From the GEPIA platform, we can see that steroid SULTs have a wide distribution throughout human body both in normal and cancer tissues (http://gepia2.cancer-pku.cn). Although ELISA kits for detection of plasma steroid SULT are available, there has been a dearth of studies focusing on plasma SULT level in cancer patients. Our findings therefore serve as the rationale for further testing plasma SULTs in ccRCC patients.

The steroid sulfotransferase enzymes that belong to the sulfotransferase family, are cytosolic enzymes that use 3′-phosphoadenine-5′-phosphosulfate (PAPS) as a sulfate donor in the steroid metabolism. SULT1A1, SULT1E1, SULT2A1, and SULT2B1 have been established as four steroid sulfotransferases. SULT1E1 and SULT1A1 have high affinity for estradiol as substrate while SULT2A1 prefers DHEA as substrate. Notably, the substrate of these sulfotransferases is not single but mutually connected [5].

The sulfotransferases are such basic and indispensable enzymes in metabolism that they may participate in the originality and development of cancer. The heparan sulfate sulfotransferase 3-OST3A (HS3ST3A) were reported to catalyze the final maturation step of HS and the abnormal synthesis and processing of which plays a prominent role in tumorigenesis [11]. Expressions of Chondroitin-4-sulfotransferase (CHST11) as well as other CHSTs in ovarian cancer samples are significantly higher than that in non-malignant ones, indicating poor prognosis [12]. Another study demonstrates targeting SULT2B1b may enhance the sensitivity and efficacy to TNF treatment in prostate cancer [13].

Thus far, there has not been report focusing on role of steroid SULTs in ccRCC. Our findings in part elucidate the prognostic contribution and functional analysis of this gene set. Given the reproductive nature of this study, several findings warrant external validation which are now in progress. However, based on robust genomic data from several public datasets, speculations can be drawn from the present study. First, overexpression of steroid SULT genes are likely to be reactive in the first place given that the set of genes are not significantly upregulated in cancer tissue of kidney as compared to normal tissue. That overexpression being the sole type of alteration throughout over 500 cases also supports the notion. Second, we speculate this gene set represents a unique genotype in ccRCC. Loss of 9p which features CDKN2A deletion has been established to confer very poor prognosis in ccRCC and this population harbors a series of other genetic alterations that may be secondary to the chromosomal alteration [14]. Lack of overlapping population between our genotype and 9p loss indicated that reactive SULT gene overexpression may originate from other underlying genomic alteration. Given that several massive comprehensive genomic databases of ccRCC are now released including the TRACERx project [15–17], probing for the attributive genomic event for this genotype is also in progress by our group. Kidney cancer is characterized with immune exclusion and novel immune checkpoint blockade (ICB) has thus become the mainstay of treatment for metastatic disease [18, 19]. Thus far, cancer-intrinsic pathways that mediate immune exclusion in ccRCC has only been reported in limited studies. Our findings give rise to another possible mechanism how immune cells are excluded in ccRCC [20]. Last but not least, our findings have the implication of developing novel prognostic biomarkers in ccRCC. Based on the several cutoff values we discovered in the current study as well as absolute expression scores obtained via RNA seq in paired samples, it is possible to integrate genetic score of steroid SULT genes into nomograms for survival prediction.

## MATERIALS AND METHODS

### Sulfotransferase gene set

The gene set of sulfotransferase family was defined by BioGPS at http://biogps.org. Using search term of “sulfotransferase”, we identified 64 annotated SULT genes in human. Four steroid sulfotransferases including SULT1A1, SULT1E1, SULT2A1, and SULT2B1 were evaluated for mRNA expression, which were further categorized into: “Steroid Sulfotransferase mRNA Altered” and “mRNA Unchanged”.

### Data processing

All data of gene mutation, CNVs, expression and protein of patients with kidney renal clear cell carcinoma were acquired from The Cancer Genome Atlas (TCGA) [21]. Genetic data of 446 tumor samples were analyzed using cBioPortal (at http://www.cbioportal.org) to help identify expression patterns and survival correlations. The two expression categories were compared for overall and disease-free survival [22, 23]. The mRNA expression level (TPM) was inferred by RNA Seq V2 RSEM and was used in patient survival via log-rank scale. The immunohistochemistry analysis between normal and carcinoma tissues were obtained from The Human Protein Atlas (HPA) at  https:www.proteinatlas.org. The GEPIA and UALCAN platforms were used to profile expressions of genes between normal and cancer tissues of kidney (http://gepia.cancer-pku.cn/ and http://ualcan.path.uab.edu/index.html) [24, 25]. Continuous data were analyzed using Wilcoxon Rank-Sum test while categorical data were analyzed using Chi-square test. Enriched genes generated from the cBioPortal platform were processed using the NET-GE dataset as the probe of functional annotation (http://net-ge.biocomp.unibo.it/enrich) [26, 27].

### Evaluation of the Immunological Infiltrate

The immunologic infiltration data were collected from the TIMER (Tumor Immune Estimation Resource, https://cistrome.shinyapps.io/timer/) platform to explore the correlation between sulfotransferase genes. TIMER is a comprehensive resource for systematical analysis of immune infiltrates across diverse cancer types. The abundances of six immune infiltrates (B cells, CD4+ T cells, CD8+ T cells, Neutrophils, Macrophages and Dendritic cells) are estimated by statistical method mining sequencing data retrieved from TCGA, which is validated using pathological estimations. [28, 29]. The major module was designed to explore correlation between gene expression and abundance of immune infiltrates. When genes were input, the scatterplots will be generated showing the purity-corrected partial Spearman’s correlation. Genes highly expressed in the microenvironment were expected to have negative associations with tumor purity, while the opposite was expected for genes highly expressed in the tumor cells.

### Gene set enrichment analysis (GSEA)

The GSEA-3.0.jar software was downloaded and gene sets (“c2.cp.kegg.v6.2.symbols.gmt” and “c7.all.v6.2.symbols.gmt [immunologic signatures]”) from the website of Broad Institute were retrieved and run under the support of Java 8.0 [30]. We considered cases with high mRNA expression level (z-score threshold: ±2) as group “steroid SULTs altered” and the rest as “steroid SULTs unchanged”. 20440 genes were involved in the enrichment process.

### Cell lines and viral infection

The 786-O and A498 ccRCC cell lines were obtained from cell bank of Chinese Academy of Science (CAS) and maintained in DMEM medium supplemented with 20% of calf serum. The human SULT1A1, SULT1E1, SULT2A1 and SULT2B1 cDNA clones were obtained from Origene and a lentivirus-based vector was constructed as previously reported. Infection of the cells was performed at 100 MOI. Transcripts for shRNA construction targeting each of the 4 genes, were selected from the The RNAi Consortium (TRC, https://www.broadinstitute.org/rnai/public/). Vectors with resistance to puromycin were constructed and transfected via non-lipofectamine Fugene transfection. After incubation, medium was replaced with complete medium supplemented with 1:5000 of puromycin and changed every 3 days until all clones were negative for ENO2. Efficacy of infection was detected using quantitative PCR (Q-PCR). The primers were designed using PrimerBank. Reactions were run using the SYBR Green Premix system on an ABI 7500n system.

### Flow cytometry

Cell cycle and apoptosis were detected using flow cytometry on a FASCanto System. For cell cycle analysis, cells were first rinsed and fixed with chilled ethanol. Cell cycle staining buffer was then applied and cells were processed on FASCanto. For apoptosis, cells were harvested and treated with Annexin V and PI. Apoptosis was designated as the sum of early and late apoptotic cells.

### Proliferation assay

Cell proliferation was studied using the MTT Cell Proliferation and Cytotoxicity Assay Kit following manufacturer’s protocol. Briefly, cells cultured at 24 h, 48 h, and 72 h were processed with MTT reagent and were then detected on a plate reader.

### Cell invasion and migration

Inserts of Transwell 24-well plates were processed with or without Matrigel for invasion and migration assays, respectively. Cells previously uninfected or infected with NANP lentiviral vector were resuspended seeded into the interior of inserts. The lower chambers were then filled with complete medium. After 72 h, cells invaded through the membrane were stained and observed at ×200 magnification.

### Colony formation assay

The colony formation assay was used to profile anchorage-independent growth of CRC cells. Generally, 6 mm plates were paved using mixture of 0.6% of complete medium and Nobel agar. On top of that was the mixture of 0.4% of complete medium and agar in which CRC cells were resuspended. After a fortnight of culture, plates were stained with 0.005% of crystal violet and colonies were counted microscopically.

### Statistical analysis

The data were downloaded from the TCGA and analyzed by GraphPad Prism 7 for Mac (GraphPad Software, San Diego, CA, USA). We used the GraphPad Software to perform Kaplan-Meier curve and log-rank test to find out significantly prognostic gene events and have a further discussion with Cox proportional hazard regression model, which could be multiple-factor applied. Log-rank P value for Kaplan-Meier plot showed correlation between mRNA expression level and patient survival. Infiltration of immune cells was also performed by GraphPad. For in vitro assays, comparisons between groups were analyzed with the 2-tailed Student’s t-test. All the results with P value of < 0.05 were considered statistically significant.
